# Selections and Abstracts

**Published:** 1912-02

**Authors:** 


### SELECTIONS

### MEDICAL IDEALS FOR THE EVERY-DAY DOCTOR.

W. W. Anderson, M. D., Newport, Ky.

The ideals of to-day are the history of to-morrow in the embryo.

Who better than the man of medicine may have high ideals? Who better than he may anticipate the history he is helping to make? Who may look back to the history of the past with such pride in his profession? If we may judge the future by the past, if the deeds that men have done are a guide to what they may do, if the record of other days is an inspiration for days to come, if a noble heritage obliges us to nobility, then how great is our debt to the past! How deep is our duty to the present! How lofty is our inspiration for the future!

Every great movement for human welfare in promoting health and vigor has been investigated, advocated, and carried to success chiefly by the medical profession to its own great loss, and often in the face of bitter opposition from the people who were to be benefited. The whole pathway of medical research and progress is marked by the bloody footprints of the martyrs of science—a noble martyrdom as lofty as any the church can boast. Vesalius was persecuted because he dared to study the dead body that he might know and heal the living. Jenner was ostracized and pictured as a devil incarnate. Harvey was decried as an infidel. McDowell was threatened with criminal prosecution. Jeffries was mobbed. Flexner is branded a monster. Carroll, Lazear, and others, died to prove the cause and secure the prevention of yellow fever, and the government took twelve years to grant a widow's pension for the service.

No other calling or profession has such a record of constant and unselfish devotion to humanity. When the great Judgment Day shall come, Medicine may stand erect and humbly unashamed, while even the Law and the Church must hang their heads, for Medicine has always been the friend of the people. The Law, designed to safeguard the liberties of man, has often riveted the fetters of his slavery; and the Church, ordained to give men that highest truth which shall make them free, has at time so far forsaken her high behest as to declare the cruel yoke of the oppressor holy and heaven-sent. No such charge can be sustained against the healing art.

Does someone say "How about the quack doctor?" How about that insatiate vampire that lives by the blood of its victims—that unmitigated villain who robs the foolish and the suffering of money and of life itself? Are we charged with his deeds? Better charge Christianity with the hypocrite. For have not we cast out the quack? Have we not ostracized him? He is none of ours. And when we have done with him, has not the protecting arm of the Law been thrown about him and is he not received into the bosom of the Church? Does he not sit in the congregation of the saints, and do not even your religious papers befoul themselves with his lying and disgusting advertisements? Have not the courts again and again given him the advantage of every known technicality and quibble that he might not have impaired his most sacred liberty of fleecing the people? No, he is none of ours. Some of us have been regarded as unorthodox in religion because we found difficulty in believing in a burning hell; but we are coming around all right. We had to put fire and brimstone back into our creed, for we had no other suitable way of finally disposing of the quack doctor.

What is the spirit that animates the medical mind and must always animate it from time immemorial unto the end of history? Not the greed of gold. The average annual income of Kentucky physicians (and they are as good as others) is less than $1,000. There is hardly a doctor anywhere in the profession who could not double his income if he but adopted a code of morals that is daily in practice and accepted in the business world. Not the fanfare of fame. Even the greatest among us will be overtaken by his epitaph long before his name is heralded among the people. Not the pride of power, for such power as the doctor attains is exercised for the public good, hampered by political and personal indifference or hostility. Ask Dr. Wiley about power. And yet Medicine goes on healing and preventing diseases, in the main unmindful of money, forgetful of fame, and puny in power.

Do you remember Him who looked down from the slopes of Olivet on the holy city, and wept, and how He stretched forth His hand and said: "Oh! Jerusalem, Jerusalem, thou that killest the prophets and stonest them that are sent unto thee, how often would I have gathered thy children together even as a hen gathereth her chickens under her wings and ye would not!" Those were the words of Him who was known as the "Great Physician." If the humane and benevolent spirit that pervades the medical profession could be gathered into one great personality, then would that great beneficent soul of Medicine look down on the suffering world and weep, saying: "Oh, erring sick, and sorrowing humanity! How have I longed to bestow on you the boon of health and vigor and life abounding and ye would not!"

Your call to the service of the healing art is not the call of wealth, or fame, or power, or popular favor, or personal aggrandizement, or social distinction, or ease, or comfort or long life. It is a call to war; a life-long enlistment on the side of humanity in its grim struggle with disease and death.

Man enters the world wailing and leaves it with a moan, and punctuates the journey from the cradle to the grave with many a cry of distress. He who deftly launches the traveler on the sea of life, who safely pilots the voyager through its storms and stress, making him fit for his seamanship until his bark is gently beached on the shores of eternity, has a great office to fill. So to conduct the advent of a new life as to make it safe in itself and free from damage to its maternal source is an important duty. To act as the counselor and guide of that new life in all matters physical, and to have a large share in shaping its mental and moral destiny, is a great task. To marshal the vital forces of that life and take command in its fierce battles with disease and death is a large responsibility. So to lead that life as to free it from self-destroying follies and lift it above foolish fears and superstitions, clearing the way for the development of its best powers and promoting its efficiency, is a lofty labor.

This is the work of the every-day doctor and it is a man's work, worthy of great men for its doing.

Man is never born great except potentially; nor can true greatness be thrust on him. It can be attained only by achievement. The man who habitually and heartily measures himself against the task before him is sure to

> "Grow each day and hour

> Into his life's potential power."

And the limits of that power, even to the every-day doctor, who shall set?

Our task calls for the active, attentive use of our best powers. We are not only to perceive what is thrust on our senses, but to seek; not only to know what is forced on us by sight or hearing, or contact, but to look, listen and feel. The amount of knowledge acquired by the senses, its clearness and its permanence will depend largely on whether the senses are actively seeing knowledge o only pasrsively receiving it. The same is true of the other faculties. If we allow our minds to drift and our conclus:ons to form themselves, they will be shallow, uncertain and unsafe. The matters we are called on to judge are far too important for this. They demand that our judgments be based on broad and thorough observation, careful comparison, and sound reasoning, the result of trained faculties act vely at work. The best-trained mind is that one which, in the largest percentage of instances, automatically does its best, which does not need to be whipped up in each case to do good work, but has made thoroughness a habit. There are great differences in the original endowment and in the training of medical men, but these differences are not greater than those which result from experience. A man w'll either use and thereby improve his talents, or neglect and lose them.

The every-day practice of medicine is eminently fitted to make the most of any man if he but gives himself devotedly to the work for its own sake. Herein lies a large hope for the every-day doctor and the mediocre man. Look at the 'llustrious names that adorn the pages of medical history. It is nowhere recorded that they were born great. Their glory was not gratuitious. Their schooling was not superior. Their opportunities were not larger. But they had great problems to solve even as others. They had mighty difficulties to overcome even as all physicians. They had sore needs to supply, even as you and I. And they measured their littleness might'ly against the mighty force of ignorance, disease and death. They realized the worth of the physician's work and labored worthily. As workmen needing not to be ashamed, they lived the every-day life on a high plane. With extraordinary energy they attacked ordinary duties. With uncommon concern they cared for their common cases.

With patient persistence they solved and proved their problems. With a devotion to duty amounting to a holy zeal, they loved even their unlovely tasks and performed them with a thoroughness that lifted their common lives above the common things of life and grew in them the character that challenges the admiration of the world. Out of the every-day of life there came to the medical profession a John Hunter, an Ephriam McDowell, a Robert Koch, an Ochterlony, an Osler, a Murphy, a Mayo, and a multitude of others; and by what means? Can you imagine Laennec making a chest examination through a silk waist? Or Trousseau content with feeling the pulse and looking at the tongue? Or Sims satisfied with the surgery of his day? Or Cabot taking the patient's word for a diagnosis? Or Gorgas accepting his office as a sinecure? These are they that "waxed valiant in fight, out of weakness were made strong."

The every-day doctor may feel that they were men of very large natural ability and that we are not to be measured by them. They *were* giants, and we may be pygmies, but, mounting to their shoulders, we can see farther than they. It is not necessary that we be great: it is necessary that we be faithful. The great men of medicine did not start out to become great or famous; they simply did their work well and grew; for they had at hand the very best means of growth, even as you and I. They sat at the feet of the masters in studying their works, even as you and I are privileged to do. They touched hands with the best men of their day in their journals and associations, and much more may we. The race-long cry of suffering humanity assaulted their ears, as it comes to ours, and they set about its relief without stopping to appraise its money value or its offering of fame.

If any man in medicine will do that, the time will come when the profession, if not the whole people, will discover developed in him whatever greatness was potiential in his endowment. Only let him not be easily content with present attainment. Let him feel that he has matriculated for a life-long course in the great university of practical experience, where every patient is a study, where every symptom is to be searched out and traced to its origin and weighed as to its significance, where every examination is an exercise in skill, every operation a training in technic and every prescription a drill in sound therapeutic reasoning.

The school of experience is proverbially expensive. The patients of the thoughtless doctor will find it doubly so; for he goes on repeating the same blunders, overlooking the same observations, reaching by the same reckless reasoning, the same careless conclusions, doubtful diagnoses, and "shot-gun" and unknown ready-made prescriptions. Such a man, profitting little by experience, drifts or blunders through his own and his patients' lives from his observation of obstetric positions as "head-first" or feet-first" to the death report of "old age."

The great minds of medicine were not so made, nor did they come by chance. The practice of medicine has never been greatly raised by accident or freaks of fate, or strokes of genius. The great men of medicine were just hard, earnest and capable workers. They grew great by growing into the greatness of their task.

There is need that medicine grow into the greatness of its task. Great things have been accomplished, but greater remains to be done. There was a time when epidemic disease swept over the face of the earth like a devastating frost and human life withered before it like the flies. Well did the psalmist speak of "the pestilence that walketh in darkness" and "the destruction that wasteth at noon-day." For the tragedy of bubonic plague, small-pox, syphilis, cholera, malaria, yellow fever, diphtheria and cerebrospinal meningitis are written large in the closed chapters of history.

Medicine has done so much that we occasionally hear the opinion expressed that the doctor will soon lose his occupation entirely by reason of medical advance. That were indeed a consummation devoutly to be desired, but offering no early hope of attainment; for every great discovery in medicine has opened to the doctor a dozen doors of opportunity for every one it has closed.

Phagocytosis, so far from being a finality in resistance, has opened to view a long vista of agglutinins, opsonins, lysins, bacterins, vaccines, side-chain theories, complements, etc., and the end is not yet.

Ehrlich presents a new remedy which may cut short the prolonged treatment of syphilis, but in so doing he opens to research the great field of chemotaxis.

Lord Lister developed antiseptic surgery and so far from finishing surgery, it has filled the world with surgeons.

Medicine still has a great field for advancement. Tuberculosis has not surrendered. Measles and whooping-cough are still serious and universal scourges. Cancer remains the scandal of medicine. Gonorrhea and wild oats continue to be sown together and to yield their bountiful crops of disaster. The field of mental and nervous diseases is still largely a jungle of unknown horrors. The internal secretions are unsolved problems. Auto-intoxication is only a tattered cloak for our ignorance. Pneumonia baffles us very much as it did the doctors of ten centuries ago. The acute and chronic joint inflammations and degenerations are in a blur of uncertainty. Kidney disorders and arteriosclerosis await elucidation. There is much territory for medicine to explore and still more to conquer.

Great as is the call for the advancement of our science, the demand for improvement in our practice is still greater; for our science is eagerly and earnestly using every means at its command to solve the mysteries of the unknown, while our practice is falling far short in applying the truths already revealed.

When the science of medicine has presented a new means of accurate diagnosis, or developed a better treatment, or evolved a surer prognosis, or offered a safer prophylaxis, it would seem reasonable that these discoveriee should immediately appear in practice and rapidly become universal in use. It would appear natural that the science and the art of medicine should walk hand in hand; that theory and practice should keep step in the onward march of medical progress.

The world is right to expect as much. If my child is ill or injured, I desire that the art of medicine, as practiced at his bedside, shall apply the best that world-wide medical science has to offer, and I hear the voice of one saying, "Whatever ye would that men should do to you, do ye even so to them."

In only one department of practice is our art following closely in the footsteps of science—the department of major surgery. The other specialties are lagging noticeably, and general practice (the work of the every-day doctor) is so far in the rear as to create some doubt whether he belongs to the procession or only happens to be traveling that way.

The surgeon leads because his work is spectacular and is done in the lime-light of publicity; because it is constantly reviewed under the fairly competent criticism of the general practitioner; because it commands a fee that supplies the means of good work; and because the surgeon occupies a limited field which he can cultivate intensively and in which he can keep up to date and become highly proficient. The same reasons hold good, but to a lesser degree, in the case of the other specialists. The work of the every-day doctor is of the every-day life, attracting little notice and reviewed by no authority nearer than the recording angel, or more capably critical than his own conscience. If well-done, it is shamefully underpaid, and his field of labor is so extensive that intensive cultivation, proficiency, exactness, and keeping up to date, are impossible. Not much is exacted at the hands of the every-day doctor. His clientele does not require much and is not competent to judge whether he gives little or much. The demands of the law are small, only asking "ordinary care and diligence" and conscience is almost as easily satisfied.

But there *is* a call to higher things and eager men will harken, earnest men will hear, and honest men will heed. It is heard in the voice of scientific medicine, whose every established truth is a prophecy of good to humanity that pleads for fulfillment. It speaks in the tones of the healing art as she asks that the most beneficent task ever entrusted to human hands be well done. It comes to us in the world-old and ever-present cry of human need. It makes its mute appeal from the silent graves of the untimely dead that ask of us their rightful years cut off. It challenges us in the words of Him who said "What do ye more than others?" It upbraids us with the dupes of quackery, 99 per cent. of whom have slipped through our hands unhealed and unsatisfied. It stirs in the life of every man whose heart throbs with the joy of battle and whose mind is uplifted by the power of the ideal. What shall be the response of the profession and especially of the every-day doctor?

If we are to share in the conquests that await the advance of scientific medicine, it is necessary that we be equipped with broader knowedge, surer skill and riper wisdom. The physician is in need of a broad general knowledge of the whole of medicine. Only so can he escape the egregious blunders that grow out of narrowness of training or experience. Disease is not so exact in localizing itself or its manifestations as to warrant exclusive specialism. Every one of us should be able to see and understand far more than we can do. We may not be able to make a blood-count, or handle the *x*-ray, or use the cystoscope, or analyze the stomach contents, or remove an appendix, or definitely determine a heart lesion, but we should know the principles on which each of these procedures is based, the field for usefulness of each, and the man who *can* do the work; and that man ought to be in easy reach.

The man of widest knowledge in medicine to-day is the every-day doctor in general practice, and yet the times demand even of him that he broaden his field of vision. Even more insistently do the times demand that he narrow his field of work. Medicine has grown far too great for any one man to cover the whole field and do it well. The every-day doctor has undertaken too much and the consequence is that he cannot give, in many of his cases, the efficient service that present medical science demands. Nor can his inefficiency command the fees that the specialist receives and deserves.

The every-day doctor must do some specializing. He ought to serve his patients in every case in which he is prepared to render as good service as is available to them. If, in addition to this, he will deote himself to the special study of the chest, he will in time become the neighborhood expert in that field. If his medical conferes will do likewise in the alimentary canal, the genito-urinary tract, nervous disorders, pediatrics, general and emergency surgery, etc., each will finally become the local authority in his particular field of general practice. He will then be able to render a better service in some one line and to command a better fee because he will be worth it. His colleagues, recognizing his special ability, will call him oftener in consultation, thereby giving their patients a better service, the doctor a better income, and the profession the uplift of a better mutual understanding. At present we are confronted, in every well-populated community, with the paradox of too many doctors and too little efficiency of medical service. Let the every-day doctor, without giving up his general practice, specialize in some department of his work and this disgraceful paradox will tend to correction.

Consultations ought to be more frequent, and they would be if some near-by doctor were fitted to act as consultant. If the every-day doctor will so fit himself, he will improve his standing in the profession, increase the respect in which he is held by the public and, what is equally as important, enhance his own self-respect.

It is not necessary or desirable that the every-day doctor undertake to displace the present-day specialist; nor ought he to look with envious eyes on the opportunities and privileges the specialist enjoys, for the narrow garden spots of pecialism are well occupied and cultivated almost to the limit of productiveness, while the broad fields of general practice, now neglected, are fallow and inviting to intensive cultivation.

The greatest opportunities in the healing art to-day are waiting at the hand of the general practitioner; 95 per cent. of practice applies first to him and it is his to take what he will, his to have and to hold, if he will but prove himself worthy to have and competent to hold. Only let him not try to grasp everything, lest he show himself unable and unworthy to hold anything. Let him select so much and such kind of practice as he can serve with adequate art, commensurate with the best medical science has to offer. In cases in which his knowledge or skill are not up to that standard, let him seek aid in consultation.

or send the patient to the man who can render the necessary service.

When the every-day doctor has learned to excel himself and others in something, he will begin to experience the pleasure of leadership, the distinction of success, the joy inherent in good work well done and the profits that in time accrue to worth.

When by doing less he has learned to accomplish more, when his skill in something has made him unwilling to do anything unskillfully, when his art is treading on the heels of science, then will medical science hurry forward to new conquests and the spirit of the healing art will go forth among the people who too long have "suffered many things of many physicians" in vain, and many will touch the hem of her garment in well-founded faith and be made whole and Medicine will find her rightful place in the esteem of men. Is this too good to be true? The answer rests with you and me, for in the Providence of God and the progress of medicine, there is nothing too good to be true.—Jour. A. M. A., Jan. 27, 1912.

---

### PROPRIETARY PREPARATIONS.

The problem of modern pharmaceutical preparations is one that should receive the calm, thoughtful consideration of every thinking medical man. We say "calm, thoughtful" consideration, for the problem has been subjected to so much hysteria and hasty, intemperate condemnation that many cardinal facts have been overlooked or disregarded entirely. No honest man in studying any question wishes to lose sight of any facts or truths that can help him to form correct conclusions. Consequently it is desirable that every topic be approached in a spirit of fairness and in a state of mind that will enable one to grasp not only one but every side of a proposition. Certainly without this fair and honest method of investigating every vital question, no one can expect to arrive at a fair and honest conclusion. Unfortunately, there has been altogether too common a tendency to consider many problems in the light of personal prejudice rather than in that of justice or truth. It is a very human shortcoming to form personal prejudices or ill founded information, and as medical men are essentially human, many popular views to the contrary notwithstanding, this shortcoming has been very frequently met in medical circles. Unfortunately, again, this tendency to draw hasty conclusions on insufficient or erroneous data has led the whole profession into many embarrassing positions. We say the whole profession for it has always happened that the few most apt to condemn good things hastily, or to laud worthless things precipitately have been so placed in positions of authority or with such opportunities for expressing themselves as to appear to represent the whole profession. As a matter of fact, and this accounts for the way that the great majority of medical men have so promptly reached a sound position on most questions, those who have presumed to speak for the whole profession have rarely if ever presented other than their own views. Naturally these erstwhile leaders or spokesmen of the profession have had a certain following. The medical fraternity has its sycophants and hypocrites as well as all other callings. But, thank heaven, the average doctor to-day does his own thinking. He makes mistakes, he lacks much that he ought to have, and in his innermost self realizes his deficiences as no one else possibly can. But there is no denying the fact that the average American physician is doing better work for suffering humanity than ever before. He has made himself a better diagnostician, he is a much more accurate observer, and he gets results or at least takes his patient to some one who can, that were undreamed of twenty years back. Just witness his work in preventive medicine! Could the remarkable success of the past decade have ever been reached but for the country's faithful practitioners? With a devotion to human needs that cannot fail to command respect and admiration, the average doctor has sacrificed every selfish interest and thrown himself into the struggle against disease with but one aim, to do his duty as he saw it. Everyone who knows many doctors, country doctors especially, is bound to appreciate the nobility of their work, and realize the absolute truth of the foregoing.

These are the men who must calmly and thoughtfully analyze the pharmaceutical questions of the hour. These are the men who must consider not alone the abuses but also the benefits that attend the use of pharmaceutical remedies. Common sense and intelligence will tell them no industry could ever have reached the proportions that the pharmaceutical has unless in response to definite needs and demands. Realizing all this and omitting no relevant fact, medical men will be in a position to weigh the evils that have developed and decide sensibly and conscientiously what their attitude is to be toward modern pharmaceutical products.

We hold no brief for the pharmaceutical manufacturer in these remarks. No one who knows anything about pharmacy and its offspring, the pharmaceutical industry, can deny that grave evils and abuses have been allowed to creep into the manufacture and exploitation of the remedies which have been submitted to the medical profession. That a little learning is a dangerous thing was never better exemplified than in the evolution of many remedial preparations and their introduction to physicians. Enthusiastic and honestly confident of the virtues of a product many a manufacturer has fallen into the error of making extravagant and exaggerated claims concerning its action. As a consequence, a large amount of pharmaceutical literature has been prepared and distributed to the profession that has been ridiculous. For laymen to prepare literature for the guidance of medical men has been not only the limit of bumptiousness, but positively insulting to the whole medical profession. This, of course, does not refer to literature prepared by chemical or pharmacologic experts, nor compilations and abstracts made by men of literary ability specially trained in chemistry or physiology. Appreciating the situation, many of the reliable houses who have sought professional patronage have never allowed any literature to go to medical men that was not prepared by competent experts or at least carefully consored by some one specially qualified. Indeed, not a few firms have employed medical graduates trained in laboratory or pharmacologic work to prepare all scientific literature designed to go to the profession. It requires no argument to show the desirabiity of having all pharmaceutical booklets, circulars, etc., prepared by those competent to make correct statements and form proper and warrantable conclusions. By the same token it is quite as apparent that literature prepared carelessly or by those who are incompetent, is neither honorable nor decent. As a matter of self protection, if nothing else, one would suppose that every firm would hesitate to submit any statement or offer any advice to medical men without assuring themselves as to its accuracy and authenticity. That there have been plenty of firms who have taken no pains with their literature, except to see that it lauded this, that or the other product to the skies, has been a shame and nothing but condemnation is deserved by such methods. So much for statements based on ignorance and incompetent material. Relative to sophisticated, urwarranted and untrue statements, not a single word of excuse of extenuation can be uttered. The firm or person who for the sake of monetary gain can wilfully claim that a product offered for the relief of sickness or suffering, is what it is not, or contains what it does not, deserves no consideration at the hands of honest men. Dishonesty, no matter what its guise, is hateful and disreputable. It can claim nothing but the severest censure and criticism. Frankly and freely we do not hesitate to say that any pharmaceutical product that is dishonest and a sham, in that claims are made for it *that are known to be false and untrue,* should be shunned and avoided by every medical man. Such a statement seems almost superfluous, for no medical man of common intelligence, to say nothing of honesty, would be apt to use any remedy that he knows to be dishonest. In this day of moral progress—of a keener grasp of our moral obligations as members of the social organism—there is no excuse for dishonesty. In no field of endeavor is this more true than in the preparation and marketing of medical and surgical supplies. Honest products of definite worth are bound to live and win their deserved measure of success. Dishonest products, on the other hand, with nothing but false and misleading claims behind them, are bound to fail and sooner or later go down to oblivion. It may take time to prove the dishonesty of a product, but the ultimate result in every case is as sure as the working of a natural law. Happily, medical men have become keener, more capable of detecting fraud and consequently more critical. It behooves every pharmaceutical manufacturer, therefore, to recognize all this and meet the situation fairly and squarely. A considerable number of clean, honorable firms have—to our knowledge—long been hewing to the highest standards. These firms have nothing to fear. There are quite a few other firms, who through ignorance, unwise advice or mistaken views have committed errors in the past, but thanks to various agencies they have recognized their obligations to the medical profession and henceforth their methods will be above reproach. In plain words, they have awakened to the situation, realized their mistakes, "cleaned house" and established standards that command respect. As long as they adhere to the course they have thus set for themselves, they will merit the confidence, esteem and patronage of the profession. If they keep good faith and maintain unfailing integrity in their relations with the medical men of the country, we earnestly believe they may depend on the cooperation and support of the profession. But if they waver, make the slightest concession to dishonest methods, try to use the profession as a crutch to reach the laity, or prove guilty of betraying any confidence reposed in them—they can blame no one but themselves for whatever may happen.

It is a matter, however, for sincere congratulation that so few of the firms dealing with medical men more or less exclusively can be classed as intentionally dishonest. Aside from ordinary reasons of policy and the common sense business methods that have caused the great majority of those who manufacture and market medical and surgical supplies to adhere strictly to honor and honesty, a genuine pride in the quality and efficiency of their products has not failed to give material aid in this same direction. It is a healthy sign when a manufacturer shows pride in his preparations and their efficiency. Honest pride helps to create ideals, and ideals are as valuable in the pharmaceutical industry as they are in any other line of human enterprise. While we do not assume to speak for the medical profession, we believe that medical men generally will be quick to appreciate the growth of ideals in the development, manufacture, exploitation, and sale of everything that they can employ with advantage and safety to their patients, or special convenience and satisfaction to themselves.

In our effort to point out that much rests with the pharmaceutical manufacturers of the country in whatever successes they may win—or good they may accomplish—in the future, we have not forgotten that the medical profession has a reciprocal part to play. All pharmaceutical manufacturers have clear cut duties—so also have all medical men. On one side are obligations to be honest, to tell the truth, to respect and keep inviolate all confidences, to make all possible progress, and to keep good faith; on the other are the obligations to give a fair hearing, to form no hasty conclusions, to respect honest enthusiasm and endeavor, never to condemn without considering all phases of a proposition, and finally to realize that the physician who neglects to use a product that represents the slightest advance is as false to his trust, as the physician who on insufficient data uses and praises a product that has no merit or value. All in all, the solution of the pharmaceutical problem would seem to rest on a better realization on the part of the interested parties of the honest obligations each owes to the other.

Pharmaceutical abuses unquestionably were responsible for the organization of the Council on Pharmacy and Chemistry of the American Medical Association. When this plan for the correction of abuses and evils in the pharmaceutical industry was definitely launched at the Portland, Oregon, meeting of the Association in 1905, the writer felt that it was a mistake, that it was a dangerous movement, illy conceived, and altogether much more apt to do harm than good. Many and various were the criticisms heard and it was believed by not a few that the whole undertaking was a scheme to drive the specialty manufacturers out of business and further the interests of certain large manufacturing houses. For some time these ideas—supplemented by many wild rumors—held sway, and the harsh, militant methods brough immediately into action all served to strengthen these views. But gradually our opinion concerning the Council on

Pharmacy and Chemistry has undergone very great change, and a movement that we viewed with suspicion and regret, we are now free to commend. The creation of the Council was a progressive step, it was opportune, and without the slightest hesitancy we are ready to say that the project is fundamentally good. No honest man who has kept in touch with the work of the Council can deny that much has been done that deserves hearty commendation. But while granting all this, it must be freely admitted that certain criticisms are merited. The Council, with all the good accomplished, has made its mistakes and shown its deficiencies, just as all other man-made institutions always do, and the man who denies this is as great an enemy to real progress as the man who questions the benefits that have assuredly been obtained. To begin with, more than one real, highly obnoxious evil has been exposed. But not a few things brought forward as vicious evils have been mistakes that have crept in through usage and custom. Tolerance, however, has been seldom exercised, and not a single error or shortcoming has failed to receive its full measure of caustic condemnation. No consideration has been shown for errors of judgment or the mistakes attributable to carelessness or unwisdom. All this is to be regretted. If the Council on Pharmacy and Chemistry has failed, therefore, to accomplish all that was anticipated, this can only be laid to the fact that it has been too destructive in its methods and has shown too little fairness and liberality.

Much as we would like to have our remarks on the Council taken in the earnest kindly spirit they are written, we know they will be misinterpreted and looked upon as ulterior in motive. But it is a common thing to be misunderstood, to have one's unselfish and honest efforts viewed askance. So we must accept the practical certainty that we shall be charged with selfish purposes. with malicious efforts to belittle the work of the Council and with being an enemy to the Association and what it is doing.

Right here let us say that this is one of the principal criticisms we have to offer to the Council and those who are directing the work of the Association. Every adverse remark, every objection, every honest expression of doubt immediately damns the individual and wins him the designation of being an enemy to the Association. It is apparently inconceivable that any person can be thoroughly appreciative of the great good that has been accomplished and still see simple details that arouse honest criticism. As we have repeatedly said, few institutions in this great country of ours are as deeply admired and respected as the American Medical Association. If the whole American medical profession felt as the writer does and appreciated as sincerely the genius of those who have made the Association the force it is to-day in American medical affairs, every physican in the country would be a member of the Association and a reader of the Journal. The work that has been done in a few years has been remarkable. From an insignificant body made up of a few thousand physicians, the Association has grown under intelligent guidance until it is one of the largest and most powerful organizations in the world. Let no one make the mistake of thinking that the wonderful progress the Association has made has been spontaneous or has resulted from the natural course of events. Hard, conscientious work by a few men who have toiled faithfully and well has brought the Assoication to where it is. All credit to such work and those who have given so much, cheerfully and uncomplainingly. We would be recreant indeed to every decent instinct if we denied one iota of the honor and crdit that belongs to the men who are responsible for the Association's present commanding position.

But having acknowledged the good things, we are entitled to make such criticisms as we deem essential to those details that bid fair to hamper the progress that we and all other earnest members hope and expect to witness.

First is this attitude of intolerance to criticism however kindly tendered. No man or group of men has a monopoly of knowledge, morality or virtue. Every one is open to mistakes and misunderstandings. So in the work of the Council on Pharmacy and Chemistry, conclusive as some of it has been, a large part of it has been opinion, conjecture and belief, and therefore essentially controversial in character. Without the slightest equivocation, we deprecate the nihilistic note that has been so prominent in the comments and conclusions of the Council. It should be remembered that for years *a large part of the income of the Journal came from the same firms whose products are now so severely condemned.* Without the financial support of these firms, once so eagerly sought and so freely accepted, it is doubtful if the present surplus would have reached its handsome proportions. It does not seem right or proper that the sudden decision of any group of men, however, erudite, should be the sole determining factor as to a product's worth or uselessness. If a preparation or a number of preparations have been fit to use for many years, without the slightest question as to their quality or efficacy, does it seem reasonable that on the instant say-so of a few men they become valueless, lose every virtue and become harmful to employ? Such a conclusion is little short of ridiculous, and while it was eminently proper to establish fixed standards which each and every product should conform to to secure admission to the advertising pages of the Journal, to make failure to conform to these standards the sole reason for discontinuing the use of a product does not leave very much to a physician's knowledge or intelligence. If a preparation really deserves such sudden rejection, one may be very sure that few intelligent medical men have been employing it for a long time, while on the other hand, if physicians have been using a product right along with more or less satisfaction, its sudden rejection is pretty apt to be unfair and unwarranted. The point we would make is that the standards of the Council are arbitrary rules laid down by the pharmacists, chemists, laboratory experts, and non-practitioners of medicine, who constitute the Council. These standards as a result are debatable, their very origin makes them subject to controversy, and the physician who denies their accuracy in toto has as much right to his opinion as the one who accepts them without question. Dragging in the National Formulary "substitutes" for original preparations was a grave mistake, for many a physician has asked with all propriety how, if some product advertised for years in the Journal must be suddenly rejected, it can be wiser, safer or more ethical for him to employ a very palpable substitute? To state it as it appear to us it all depends on the view point of the individual doctor. If a physician prefers to use a "substitute" for any original product or preparat on, that is entirely a matter for him to decide. He is the arbiter of the question and the responsibility of the decision rests on him alone. After all that can be said concerning these various matters, the fact that persists is that *the individual physician is the court of last resort.* He is the one to decide what he can or can not, will or will not employ, in his work. It is right for the Journal to supply him with all the information available that will help him to arrive at correct conclusions concerning the value of different preparations. The Council of Pharmacy and Chemistry is essentially an investigating or examining body whose proper province is to ascertain and present facts concerning all pharmaceutical and drug products, everything indeed that is employed in the treatment of disease. The more accurate, definite and unprejudiced such information is, the more helpful it will be and the greater aid it will offer in solving the pharmaceutical problems, which, after all, are only incidental to the great, big, burning question that confronts the profession as never before, *how best to utilize every useful agent with greatest degree of safety and success.*

If the Council on Pharmacy and Chemistry will thus direct its efforts to the broader questions of clinical therapy it is certain that the Association will do a work not only for its members, but for all humanity that can not fail to bring results of the most far reaching character.

The recent attack on the American Journal of Surgery serves to emphasize all the evils and dangers of a policy of destruction and ruin. Athough freely admitting the worth and high character of the reading pages of this publication, the Journal nevertheless condemns it in the most severe terms, hotly taking to task all those who contribute to its reading pages, and all because it carries the advertisements that only a short time ago were also freely admitted to its own pages. A long list is given of products that make the American Journal of Surgery, according to the Journal of the A. M. A., a dangerous publication and one that in spite of its meritorious reading pages medical men should avoid, boycott and turn from generally. We have too great faith in the American physician to believe he will tolerate such unfairness. With a few exceptions the worst that can be said against any product in the list of those advertised in the American Journal of Surgery, is that it has not been approved by the Council of Pharmacy and Chemistry! To make this "approval" the touchstone of honesty and therapeutic efficiency, is not only ridiculous, it is a perversion of right, and if it prevails will offer the greatest impediment to therapeutic progress that has ever appeared. *Will the thoughtful physician accept without question the dictum that he can not honorably or honestly employ any drug or remedial preparation that has not been "passed" by the Council of Pharmacy and Chemistry of the A. M. A., a corporation of the State of Illinois?* Will he refuse to read any journal that appeals to his intelligence and interest simply because its advertising pages carry announcements concerning drugs and products that have not been accepted for inclusion in the book published by the Council and known as New and Non-Official Remedies? We doubt very much if the rank and file of the profession will submit to any such domination, for no intelligent physician can fail to see that valuable and useful as the work of the Council may be, they are none the less open to controversy and change. The preposterous idea of taking our therapeutic morals and practice from the Council's deductions exclusively is apparent from more than one standpoint. For nearly seven years the Council has been at work and yet the last two years has witnessed the acceptance of several well established products. Can it be believed that any intelligent physician has been kept from using such products by the tardiness of the Council to accept them? Again not a few useful preparations have had their acceptance delayed by prolonged controversies as to some real or fancied fault in their nomenclature, labels or literature. Months in many instances have passed before these details have been arranged to suit the Council. Not a doubt has existed relative to the therapeutic efficiency of these products.

*Should the physician have denied himself the service of these remedies while they were held up solely by technicalities?* Finally, quite a goodly number of products that have enjoyed the Council's approval, and graciously been allowed to advertise in the Journal have recently been cast out into outer darkness. For one reason or another such as objectionable name, uncertain utility, and so forth, these preparations are no longer acceptable to the Council. In passing it may be remarked that they are no different than they were at the time they were "passed" and admitted to the inner circle. In connection with their original acceptance they conformed to every requirement and made all changes demanded of them. But alas, the Council is revising its standards, altering its opinions, and viewing matters in a far different light than at the outset for products supposedly fixtures in New and Non-Official Remedies have been rudely and ignominiously withdrawn from the book. While these acts of the Council may be praiseworthy in that they show an admirable independence, the *all important fact for the practitioner and every interested person to note is that the Council's conclusions are by no means absolute.* Its work shows beyond all doubt that the whole problem is ambiguous, that there are no fixed values and that standards are essentially open to change. All this means what we have repeatedly said that *the whole question is in its controversial or debatable stage.* Until ideas and opinions are more settled and standards more certain and fixed, the Journal outrages every instinct of fairness and right when it makes an onslaught such as that on the American Journal of Surgery. Its conversion and "redemption" are too recent and its vaunted surplus represents too much of the same money it accuses other journals of seeking, to justify a "holier-than-thou" attitude toward a publication that is serving the medical profession as faithfully as is the American Journal of Surgery.

Our position is just this: We are thoroughly appreciative of the good features of the work the Council has been doing. But we are as sensible of its failures and shortcomings as we are of its successes and unquestionable possibilities. It would be a calamity for the Council to discontinue its work. As an examining and investigating body it can supply the profession with facts of the utmost value. But having supplied those facts its obligations cease. *It is up to the medical profession to weigh these facts, study them intelligently and liberally, and then be guided by them just so far as each man's individual judgment dictates.* We have found some, yes, much of the Council's findings of the utmost help. Other findings have left us in doubt, while not a few of their deductions have seemed wholly and absolutely wrong. As repeatedly stated we have regretted the too great prevalence of the spirit of intolerance, as we have also the too great tendency to consider all criticism as born of enmity and animosity. We have regretted that instead of fostering friendly cooperative relations with the independent medical press of the country, the apparent object has been to belittle every effort and question every motive. American Medicine does not have the slightest intention of restricting its advertising pages to only such products as are in good odor with the Council of Pharmacy and Chemistry. The medical men responsible for American Medicine are perfectly able to establish Standards that will meet every requirement of the honest physician. The pharmaceutical problem is subject to too much controversy and there is too much uncertainty on every side to justify any medical publication, aside from the Journal and possible the State Journals, accepting *the arbitrary dictum of a body that freely admits it may repudiate to-morrow the action it takes to-day!!* Again, too many of the findings of the Council have shown the bias of individual members, and too much happens to be known concerning certain of its "passes" as well as its "rejections" to warrant American Medicine—or any other independent journal—accepting them without question.

There is a mistaken idea that medical journalism is exceedingly profitable. This is indeed wrong and no independent medical publication run in behalf of its readers can ever become rich and opulent. This is borne out by every one of the high-grade journals that are honestly and fearlessly contributing their part to the uplift and progress of contemporary medical journalism. But happily there are other rewards than those measured in dollars and cents and we know we are stating the exact situation when we say that every physician or group of physicians at the helm of every independent medical journal that is justifying its existence seeks something prized a great deal more highly than money. This something is the knowledge of faithful service, a realization that one has been a factor in a constructive undertaking, even though it may have been small and insignificant. The faith and confidence we have in the American doctor, and an unfaltering belief that right always wins in the end, leave no doubt as to the ultimate outcome. American Medicine is going on striving to serve its readers and the medical profession to the limit of its opportunity and the ability of those directing it. Honesty, integrity and a suqare deal in every transaction shall be our constant aim. Service, clean, honorable service, shall continue to be our watchword. In every way we shall conscientiously strive to protect our readers against imposition, fraud or dishonesty not only in our reading pages but equally in our advertising pages. We shall exercise our discretion as to accepting articles which mention pharmaceutical products. *The slightest suspicion of co lusion between an author and a firm will be sufficient to lead us to take the most drastic action.* On the other hand, if a physician whose integrity is unimpeachable sees fit to mention a preparation, we shall not insult him by cutting out such mention. It matters not whether his mention is favorable or derogatory. The author is the one responsible for his statement and the journal has nothing to do with it after assuring itself as far as poss'ble that the statement is a clean and honest expression of opinion. Pharmaceutical manufacturers have ever been ready to criticize a journal that refused to allow physicians of even the highest standing to mention pharmaceutical products by their proper names. That we henceforth leave the matter to the discretion of each author will probably gratify those manufacturers who have complained so bitterly when the names of products have been eliminated by a zealous editor. But in this connection we want it distinctly understood that adverse opinions will likewise have no restrictions. If a manufacturer wants a journal to be independent and liberal enough to print the full and literal statement made by any honorable physician who may chance to speak favorably concerning any honest pharmaceutical product, he must be big enough to acknowledge the justice of printing no less readily the comment of the honest physician who may happen to speak unfavorably. This is simply in line with American Medicine's efforts to merit not only the respect but also the confidence of every honorable medical man. Those who guide its course and direct its policies have no doubt that mistakes will be made in the future, just as they have been made in the past. But we shall make as few as possible, and try our best never to make the same one twice. In closing, let us say that we regret exceedingly having devoted so much space to these matters of policy and purpose. We feel, however, in view of the attitude of the Journal of the American Medical Association toward the independent medical journals of the country that it is due to the thousands of honorable physicians who have given American Medicine their support during the past five years, to come out in the open and state our position exactly as we understand it. Having done so, we are perfectly willing to leave the verdict to the physicians of America. We brook no fight with any one. The victories we hope to win are those of honest, constructive endeavor. That there is a place not only for American Medicine but a goodly number of other purposeful medical journals, is proven by the success and standing that so many have achieved. Every one of the reputable independent medical journals of the country would be glad to cooperate with the great official organ of the American Medical Association. Every one of these journals would be glad to work along consistent lines for the abolition of every well defined evil that threatens the usefulness of the medical profession. Every one of these journals would be glad to have the splendid resources of the Association employed to point the way to better things. But not one of these journals wants to see this work, however urgently it may be needed, undertaken with a spirit of intolerance, animosity or revenge. Not one of these journals will suffer coercion, *especially by those who know nothing of the problems of the*

*practicing physician.* And finally not one of these journals is ready to concede that the establishment of a series of arbitrary rules and regulations gives the Journal of the American Medical Association, or any other journal however estimable, a monopoly of virtue, aspiration or morality. The opportunity to unite the medical profession, to marshal every active force, and to make the whole American profession a genuine fraternity is within the immediate scope and power of those who are at present in control of the Association. The opportunity is passing. It may never come again. Will it be seized and taken advantage of? Are those in command able to grasp the situation and create a united, harmonious and splendidly organized profession that shall have its destiny in its own hands, and not as our British brethren now find theirs—in the talons of the politicians? We can not say. We do not know. We can only hope. But this we can say with all the earnestness at our command and a thorough appreciation of the gravity of existing conditions, and that is aspiring medical men expect something a great deal more uplifting from their leaders than an acrimonious warfare on the independent medical journals of the country.

A little patience, and the few evils that still exist will be overcome. Permanent and lasting reform always takes time. Vast improvement has been made by every "worth while" independent medical journal and the whole movement is upward and forward. But if warfare is the program and annihilation the object, as is presaged by the attacks already made, one thing is certain, it will not be a one-sided struggle. And after the smoke and noise of battle has cleared away, one fact will stand forth and that will be that those who prosecute a holy war need to be all that they claim, or they may have to pay a cost in damaged reputations and broken halos that will be out of proportion to the victory won.—Ed. Amedican Medicine, Jan., 1912.

